# Attitudes towards the integration of smoking cessation into lung cancer screening in the United Kingdom: A qualitative study of individuals eligible to attend

**DOI:** 10.1111/hex.13513

**Published:** 2022-05-05

**Authors:** Samantha Groves, Grace McCutchan, Samantha L. Quaife, Rachael L. Murray, Jamie S. Ostroff, Kate Brain, Philip A. J. Crosbie, Janelle Yorke, David Baldwin, John K. Field, Lorna McWilliams

**Affiliations:** ^1^ School of Health Sciences, Manchester Centre for Health Psychology, Division of Psychology and Mental Health, Faculty of Biology, Medicine and Health University of Manchester Manchester UK; ^2^ Wales Cancer Research Centre, Division of Population Medicine, School of Medicine Cardiff University Cardiff UK; ^3^ Centre for Prevention, Detection and Diagnosis, Wolfson Institute of Population Health Queen Mary University of London London UK; ^4^ Academic Unit of Lifespan and Population Health, Faculty of Medicine University of Nottingham Nottingham UK; ^5^ Memorial Sloan‐Kettering Cancer Center, Behavioral Sciences Service New York New York USA; ^6^ LydiaBecker Institute of Immunology and Inflammation, Division of Immunology, Immunity to Infection and Respiratory Medicine The University of Manchester Wythenshawe UK; ^7^ Christie Patient‐Centred Research, Division of Nursing, Midwifery and Social Work, The Christie NHS Foundation Trust The University of Manchester Manchester UK; ^8^ Department of Respiratory Medicine Nottingham University Hospitals NHS Trust Nottingham UK; ^9^ Institute of Systems, Molecular and Integrative Biology, Molecular and Clinical Cancer Medicine, Faculty of Health and Life Sciences University of Liverpool Liverpool UK

**Keywords:** cancer prevention, lung cancer, lung cancer screening, qualitative, smoking cessation

## Abstract

**Introduction:**

There is limited research exploring how smoking cessation treatment should be implemented into lung cancer screening in the United Kingdom. This study aimed to understand attitudes and preferences regarding the integration of smoking cessation support within lung cancer screening from the perspective of those eligible.

**Methods:**

Thirty‐one lung cancer screening eligible individuals aged 55–80 years with current or former smoking histories were recruited using community outreach and social media. Two focus groups (three participants each) and 25 individual telephone interviews were conducted. Data were analysed using the framework approach to thematic analysis.

**Results:**

Three themes were generated: (1) bringing lung cancer closer to home, where screening was viewed as providing an opportunity to motivate smoking cessation, depending on perceived personal risk and screening result; (2) a sensitive approach to cessation with the uptake of cessation support considered to be largely dependent on screening practitioners' communication style and expectations of stigma and (3) creating an equitable service that focuses on ease of access as a key determinant of uptake, where integrating cessation within the screening appointment may sustain increased quit motivation and prevent loss to follow‐up.

**Conclusions:**

The integration of smoking cessation into lung cancer screening was viewed positively by those eligible to attend. Screening appointments providing personalized lung health information may increase cessation motivation. Services should proactively support participants with possible fatalistic views regarding risk and decreased cessation motivation upon receiving a good screening result. To increase engagement in cessation, services need to be person‐centred.

**Patient or Public Contribution:**

This study has included patient and public involvement throughout, including input regarding study design, research materials, recruitment strategies and research summaries.

## 1 INTRODUCTION

Successfully stopping tobacco smoking is the most important behaviour change required to reduce lung cancer risk and mortality.^1^ In the United Kingdom (UK), the prevalence of quit attempts has decreased since 2007.^2^ Individuals from deprived communities have the highest smoking prevalence and disproportionately worse health outcomes. For example, Manchester and Liverpool, two areas in the North of England within the most deprived decile in England, have the highest premature lung‐related mortality rates in the country.^3^, ^4^

Research has demonstrated that low‐dose computed tomography scan (LDCT) screening detects early‐stage disease and reduces lung cancer‐specific mortality in high‐risk individuals.^5^, ^6^, ^7^, ^8^, ^9^ In trials such as the National Lung Screening Trial, individuals were classed as high‐risk if they had 30 pack years and had smoked within 15 years.^5^ As such, lung cancer screening programmes have been implemented in some countries, including the United States of America (US) and China, while the UK National Health Service has funded a ‘Targeted Lung Health Check Programme’ (TLHC) following the success of multiple pilot projects.^7^, ^10^, ^11^, ^12^ The TLHC is running in areas of England with high lung cancer mortality^13^ and inviting 55–74‐year‐old individuals who have ever smoked to a free face‐to‐face, telephone or video appointment. Here, the attendees' risk of lung cancer is calculated using set questions, and in some cases, noninvasive tests such as spirometry are performed. If an individual is above a designated risk threshold, they are invited for an LDCT scan at a local screening facility.^14^

During the TLHC appointment, an attendee may be offered a smoking cessation intervention; however, there is currently no standardized approach for provision across facilities in England. Lung cancer risk assessment, and the screening process itself, may trigger a cessation‐related teachable moment: a point in time when an individual has increased desire to change behaviour^15^, ^16^, ^17^ and greater receptivity to cessation support. Indeed, screening attendance as part of a research trial has been associated with increased cessation compared to usual care, particularly among attendees who receive a positive scan result.^18^, ^19^ Best available evidence suggests that these differences can be maintained for 5 years.^19^ This success has been replicated in a UK community setting where 55% of attendees who made a successful quit attempt in the year after screening, attributed this to participation in the TLHC.^20^ Implementation of smoking cessation is additionally associated with increasing the cost‐effectiveness of lung cancer screening.^21^, ^22^

Quantitative and qualitative evidence in the US has demonstrated that screening eligible individuals believe offering cessation support as part of lung cancer screening is appropriate.^23^, ^24^, ^25^, ^26^ A limited number of studies have explored attitudes in the lung cancer screening eligible population in England. Screening results have been identified as either promoting cessation through giving participants a ‘clean slate’ if clear or by abnormal findings indicating a need to stop.^27^, ^28^, ^29^ However, research has also highlighted concerns within the lung cancer screening population, including fatalism^29^ or the belief that a clear result may not always be motivational and could promote smoking continuation if evidence‐based communication approaches are not implemented.^28^ Furthermore, those eligible for lung cancer screening have long‐term smoking histories, often since childhood, and are therefore likely to have a higher dependence on tobacco and greater difficulty with cessation requiring evidence‐based support.

The Theoretical Domains Framework (TDF)^30^, ^31^ is an integrative model comprising 14 domains related to behaviour change: for example, emotions, social influences and beliefs about capabilities. The framework was specifically developed for implementation research and has demonstrable utility for aiding exploration of barriers and facilitators to engagement in smoking cessation interventions.^32^, ^33^ It has also been used as a tool to develop several smoking cessation interventions.^34^, ^35^ Therefore, the TDF is well suited to underpinning exploration of attitudes and preferences for smoking cessation provision, allowing researchers to identify clear targets for tailored intervention development with the aim of successful behaviour change.

The protocol for TLHC,^14^ alongside a European position statement,^36^ recommends that smoking cessation should be incorporated into lung cancer screening. However, no further guidance on optimal implementation or delivery has been disseminated. Gaining insight from stakeholders rather than relying solely on published literature, which is still limited given the novelty of lung cancer screening, will aid the development of UK‐based guidelines that consider the wider contextual factors affecting implementation. Therefore, the aim of this study was to understand attitudes and preferences regarding the offer and provision of smoking cessation at the time of lung cancer screening, from the perspective of those eligible to attend.

## 2 METHODS

### 2.1 Design

A qualitative design involving focus groups and semi‐structured interviews was used to explore individuals' opinions of smoking cessation provision at lung cancer screening, and preferences for cessation support delivery. Data collection began in February 2019 and was adapted from focus groups to telephone interviews from March to August 2020, due to the COVID‐19 pandemic. Virtual focus groups were not used due to limited computer access highlighted by previous participants. This study adopted a limited realist approach, assuming meaning can be shared across participants, with potential relevance to wider populations (realist ontology), while acknowledging that participant and researcher experience is inevitably shaped by context (constructivist epistemology).^37^

### 2.2 Participants

Inclusion criteria were based on eligibility for lung cancer screening: (a) aged 55–80, (b) who currently smoked, or had quit within 3 months before study participation date and, (c) lived in one of four areas where lung cancer screening was ongoing during the data collection period. The quit period of 3 months was specified to facilitate participant recollection of their experience as a person who smokes, alongside their current experience of cessation. Three months has also been used as an endpoint to measure short‐term smoking cessation.^38^

### 2.3 Procedure

A topic guide (see File S1) was developed in consultation with a lay research partner who is screening eligible. Interviews were piloted with other researchers with expertize in health psychology to assess flow, clarity and prompts to be used. The semi‐structured topic guide was used flexibly to ensure that all topics of interest and any new views raised by participants were explored.

Participants were initially invited to take part using community outreach recruitment methods. This included visits to community locations to advertise the study to those eligible to participate in four screening‐active areas, staff members in local organizations advertising the study to visitors and disseminating paper and online posters across networks. Due to the COVID‐19 pandemic, in person recruitment was suspended, and social media advertising was adopted in March 2020. Advertisements were posted on community social media groups (e.g., residents' associations) within the same screening‐active areas. Interested individuals who then contacted the research team were provided with a participant information sheet to review by post or email.

Two focus groups were conducted in February 2020, facilitated by two researchers (S. G. and L. M.). Focus groups were held in community locations already known to participants, and participants were offered reimbursement for travel. Both focus groups consisted of only participants who currently smoked. From March 2020, solely one‐to‐one telephone interviews were conducted by one researcher (S. G.) due to the beginning of the COVID‐19 pandemic. Before each focus group or interview, researchers provided participants with information regarding lung cancer screening including the typical pathway. Upon finishing the focus group or interview, participants completed an optional demographic form (two participants did not complete the form). This included questions regarding age, gender, ethnicity, occupation, education, smoking and cessation history, desire to quit and prior lung cancer screening attendance. All participants were offered reimbursement, with the option of a £20 shopping voucher, or a Cancer Research UK donation of the same value. A debrief sheet including contact details of local smoking cessation organizations was offered to all participants.

Detailed field notes were compiled, and interviewer debrief discussions were held after each interview (S. G. and L. M.). Throughout data collection, data sufficiency was discussed. Data collection ended upon a subset of the research team agreeing the research questions had been appropriately addressed.^39^ Both data‐collecting researchers were females with postgraduate psychology training in qualitative research, and both did not smoke or have a past smoking history.

All participants provided informed consent before data collection. The study received ethical approval from the University of Manchester Research Ethics Committee (2019‐7018‐12116) and the Health Research Authority (265589).

### 2.4 Data analysis

Focus group and interview recordings were transcribed verbatim by an external transcription company. Transcripts were checked for accuracy and anonymized. Data were analysed using reflexive thematic analysis using the framework approach for data organization.^40^ Each transcript was read to get an overall sense of the data and a coding framework was developed using Nvivo‐12 software (S. G.). Where possible, themes were organized around the TDF^30^, ^31^ to assist with understanding the barriers and facilitators associated with cessation delivery preferences. Simultaneous inductive coding using no pre‐existing framework was conducted to explore wider underpinning views and experiences. The initial codebook was developed using three initial transcripts and discussed with a smaller research team to resolve discrepancies in coding and to formulate an initial framework matrix (S. G., L. M. and G. M.). The matrix was iteratively modified throughout analyses to confirm all relevant codes were captured. Data were charted into the matrix for interpretation and theme generation. Focus group data were treated as a single case in the matrix to account for the unique dynamics within each group. Participant smoking status was displayed next to each case name to allow the researchers to consider the impact of the participants' current smoking context alongside the data. The analysis focused on attitudes and experiences of screening eligible individuals regarding smoking and cessation attempts, and how this shapes preferences for cessation integration within a lung cancer screening context.

### 2.5 Findings

#### 2.5.1 Sample

Thirty‐one participants took part in this study (six took part in two focus groups, *N* = 3 in each, and 25 in individual telephone interviews). Twenty‐six participants currently smoked and five had recently quit. For additional sample characteristics, see Table 1. Focus groups and interviews ranged from 16 (incomplete interview due to participant becoming unavailable unrelated to participation, and unable to reschedule) to 69 min (median: 48 min).

**Table 1:** Characteristics of participants (A) who currently smoke and (B) who have recently quit

Pseudonym	Age	Gender	Ethnicity	Qualifications	Employment	Years smoking	Number of cigarettes or grams of tobacco smoked per day	Already attended lung cancer screening	Want to stop smoking?	Ever quit more than 3 months
(A) *Participants who currently smoke (CS)*										
Arthur	71	Male	White/White British (Irish/other)	No formal qualifications	Retired	56	5 Cigarettes	No	Yes	No
Norman	64	Male	White/White British (Irish/other)	No formal qualifications	Unable to work	10	4 Cigarettes	No	No	No
Louise	63	Female	White/White British (Irish/other)	No formal qualifications	Unable to work	50	20 Cigarettes	No	No	No
Collette	65	Female	White/White British (Irish/other)	GCSE/O‐Levels/ONC/BTEC/other	Full‐time carer/home‐maker	40	30 g Tobacco	Yes	Yes	Yes
Alan	68	Male	White/White British (Irish/other)	No formal qualifications	Retired	53	60 g Tobacco	Yes	Yes	No
Eleanor	73	Female	White/White British (Irish/other)	No formal qualifications	Unable to work	50	40 Cigarettes	Yes	No	No
Sarah	61	Female	White/White British (Irish/other)	A‐levels/higher education below degree	Employed	41	9 Cigarettes	No	Yes	Yes
Clara	Did not provide demographic details (had attended lung cancer screening)									
Julia	62	Female	White/White British (Irish/other)	A‐levels/higher education below degree	Employed	46	5 g Tobacco	Yes	Yes	Yes
Diane	62	Female	White/White British (Irish/other)	A‐levels/higher education below degree	Unable to work	3	10 Cigarettes	Yes	Yes	Yes
Anthony	68	Male	White/White British (Irish/other)	University degree	Retired	50	10 Cigarettes	Yes	Yes	No
Rebecca	66	Female	White/White British (Irish/other	GCSE/O‐Levels/ONC/BTEC/other	Employed	35	16 Cigarettes	No	Yes	No
Laurence	62	Male	Any other mixed background (White/Caribbean)	University degree	Employed	42	7.5 Cigarettes	No	Yes	Yes
Humphrey	Did not provide demographic details (had attended lung cancer screening)									
Walter	73	Male	White/White British (Irish/other	No formal qualifications	Retired	60	40 Cigarettes	No	Yes	Yes
Bernie	55	Female	Prefer not to say	A‐levels/higher education below degree	Employed	37	10 Cigarettes	No	No	Yes
Len	70	Male	White/White British (Irish/other	GCSE/O‐Levels/ONC/BTEC/other	Retired	61	20 Cigarettes	No	Yes	Yes
Catherine	71	Female	White/White British (Irish/other	A‐levels/higher education below degree	Retired	57	7 g Tobacco	No	No	No
Patricia	67	Female	White/White British (Irish/other	GCSE/O‐Levels/ONC/BTEC/other	Retired	59	25 Cigarettes	No	Don't know	Yes
Maxine	59	Female	White/White British (Irish/other	A‐levels/higher education below degree	Employed	30	20 Cigarettes	No	Yes	Yes
William	56	Male	White/White British (Irish/other	University degree	Retired	40	20 Cigarettes	No	Yes	No
Laura	72	Female	White/White British (Irish/other	University degree	Retired	30	15 Cigarettes	No	Yes	No
Helen	56	Female	White/White British (Irish/Other	GCSE/O‐Levels/ONC/BTEC/other	Employed	25	7.5 Cigarettes	No	Yes	Yes
Lee	69	Male	White/White British (Irish/other	University degree	Retired	54	10 Cigarettes	No	Yes	No
Christopher	56	Male	White/White British (Irish/other	GCSE/O‐Levels/ONC/BTEC/other	Retired	43	25 Cigarettes	No	Yes	No
Kathleen	55	Female	White/White British (Irish/other	University degree	Employed	43	20 Cigarettes	No	Yes	Yes

Pseudonym	Age	Gender	Ethnicity	Qualifications	Employment	Already attended lung cancer screening	How long quit at time Interview	Years smoked	Number of cigarettes or grams of tobacco smoked per day
(B) *Participants who have recently quit (RQS)*									
Elizabeth	60	Female	White/White British (Irish/other)	No formal qualifications	Unable to work	No	2 months	47	25 Cigarettes 5 g tobacco
Esme	77	Female	White/White British (Irish/other)	GCSE/O‐Levels/ONC/BTEC/other	Retired	No	3 months	60	15 Cigarettes
Frances	57	Female	White/White British (Irish/other)	University degree	Employed	No	1 month	22.5	2 Cigarettes
Lydia	68	Female	White/White British (Irish/other	No formal qualifications	Retired	No	2 months	49	22.5 Cigarettes
Molly	60	Female	White/White British (Irish/other	Prefer not to say	Retired	No	3 months	45	10 Cigarettes

Data are presented as three themes: (1) bringing lung cancer closer to home; (2) a sensitive approach to smoking cessation and; (3) creating an equitable service. Quotes are presented as pseudonyms with age (years) and smoking status (currently smokes [CS] or recently quit smoking [RQS]).

2.5.1.1

*Theme 1: Bringing lung cancer closer to home*

The impact of smoking on health, including its causal role in lung cancer development, was widely acknowledged by participants. Participants held complex beliefs surrounding their lung cancer risk that was characterized by two dominant yet unstable perceptions of personal risk. At times, pre‐existing health conditions such as chronic obstructive pulmonary disease (COPD) or experiences of losing others to lung cancer worried participants, and underpinned an amplified perceived vulnerability to smoking‐related illness:

As time has gone on for me, I've become more aware of my own personal health […] you've started to talk to somebody and they've told you, 'oh, did you know who was it passed away', he had, what, cancer? […] he did used to smoke a lot though, didn't he?

Arthur, 71: CS

At other times, participants discussed engaging in avoidance regarding the personal impact smoking may have. Despite worry sometimes increasing perceived risk, high levels of lung‐cancer‐related anxiety led to some participants ‘[burying their] head in the sand’ (Maxine, 59: CS), avoiding thinking about the impact of smoking on their health. Additionally, perceived good health or feeling that the consequences of smoking may never ‘catch up with you’ (Christopher, 56: CS) were also described as distancing individuals from their perceived risk of lung cancer. These views underpinned a belief that preventative measures such as smoking cessation are not yet required.

Overall, lung cancer screening was viewed by many as an opportunity to learn more about lung cancer risk, bringing the link between smoking and subsequent health consequences to the forefront of attendees' minds. This led many participants to reflect on considering cessation. Even the thought of attending an appointment specifically about lung health was suggested as a motivator, in comparison to previous discussions about stopping smoking with health professionals:

…if you just go to the doctors to stop smoking and you're given these tablets or whatever […] I need evidence of what's going on and if you […] just go to the doctors and you don't see that evidence.

Sarah, 61: CS

In the quote above, Sarah discusses the need to receive ‘evidence’ of the impact of smoking on her health. Indeed, participants reflected on the unique opportunity provided by screening to gain a personalized picture of their lung health. At times this opportunity appeared to be able to tip the balance of perceived risk, overcoming avoidance or denial that attendees may have previously engaged in. Some suggested that without personalized evidence, they would not consider quitting:

Because they're going to be a little bit more nervous because they're going to be a little bit, ‘oh, if I've got to have a scan, there must be something. You know, perhaps it's affecting my lungs.' That's what I would think anyway and perhaps be more receptive to further stopping.

Esme, 77: RQS

However, some participants remained ambivalent about the impact of risk feedback, expressing fatalistic views. Clara (age unknown: CS) described being torn between wanting to give up smoking, whilst simultaneously believing ‘it's a bit too late in the tooth now’ if her LDCT scan had returned with an abnormal finding. Despite this view being shared by other participants, discussing evidence of the benefits of cessation, regardless of age or length of smoking history, may have the potential to enhance motivation to consider cessation:

… is it going to be worth stopping if you've only got, you know, a few months to live or something like that, or whether it's just a bit of damage and it's fixable […] I saw an article […] about how the lung repairs itself now, they found that the lung repairs itself. I saw that and I thought, wow, that is amazing, if I stop smoking and my lungs repair, it's got to be good news really, that was another reason that made me want to give up.

Molly, 60: RQS

A minority of participants mentioned that if lung cancer screening displayed no evidence of damage, this may result in false reassurance regarding the negative consequences of smoking. Lung cancer screening staff discussing the potential for future worsening of lung health appeared to counteract this potential ‘licence to smoke’:

Well I hope that once you […] even though you have got the all clear, they'd show you the side effects even though you've got the all clear. Show the stuff that can go wrong if it had have been in the other scenario.

Elizabeth, 60: RQS

2.5.1.2

*Theme 2: A sensitive approach to smoking cessation*

The stigmatization of people who smoke was widely discussed both by participants who CS and those who had recently quit. Many felt that people who smoke are treated like ‘second‐class citizens’. Participants perceived having valid reasons for smoking such as a stress relief tool, which they felt are often not recognized by individuals who do not smoke. Despite acknowledging the good intentions of friends, family and healthcare professionals who do not smoke, being ‘told’ to quit often had a counterproductive impact, particularly among participants who did not wish to quit:

I mean I've been told by friends, 'well, you know, you shouldn't be doing it, you shouldn't be doing that', and I can dig my heels in more and smoke more when I'm around them.

Maxine, 59: CS

Instead of external pressure, reaching a readiness on their own terms was viewed as the most likely pathway to successful cessation. Although self‐initiated cessation was emphasized as important, a sense of feeling ‘trapped’ in a self‐described addiction was endorsed by many, alongside internalized stigma in the form of self‐blame. These feelings were intensified for some by low perceived knowledge of effective methods for quitting, or by past unsuccessful cessation attempts leading to a frustrating cycle of wanting to quit, but not knowing how, or why they cannot achieve their goal. Self‐blame and guilt had a pervasive impact on one participant, feeling they were ‘deserving’ of lung cancer:

To be honest, if at my age I don't realise the dangers that smoking can cause, then I deserve to pop my clogs with cancer.

Catherine, 71: CS

Experiences of external and internalized stigma shaped participants' opinions regarding the approach staff should take to discuss smoking and cessation during screening. Interestingly, most participants expressed a preference for support delivery from somebody who had previously smoked, and several desired the opportunity for adjunct group support to be advertised by screening staff. These preferences appeared to be underpinned by the need for a shared experience, wanting support from people who ‘know what you're going through’ (Lydia, 68: RQS) and have been through similar challenges and barriers themselves. Participants remarked that without this, support may not be as effective:

I mean, you know how annoying it is when somebody lectures you about something and you know that it's all theory. They haven't got a clue because they've never done it and they've never experienced it and they're theorising it. You know, experience works.

Laura, 72: CS

Despite a peer support system being described as ideal, some participants acknowledged that in practice, this may not always be feasible due to the small proportion of staff working within lung cancer screening likely to have previously smoked. Conversely, a minority of participants emphasized that they would specifically not want to receive support from individuals who had previously smoked. Instead, ensuring that staff members respect attendees' autonomy and empower attendees to feel in control of their decision to quit, was viewed as an acceptable and feasible minimum offer. This was emphasized by those who reported they currently did not wish to quit. The approach taken by staff was viewed as an important determinant regarding uptake of the cessation offer. In the below quote, Collette reflects on the disengaging nature of a paternal and authoritative offer, which was often expected by participants. In contrast, Walter describes that appreciating attendees' own knowledge, with a collaborative and positively framed communication approach, is much more likely to promote buy‐in:

I don't want someone in my face as soon as I walk in to say, 'stop smoking', because […] you just put the barriers up.

Collette, 65: CS

[…] ‘let's do it together, I'm here to help you. I'm not here to order you or shout at you, stop smoking, it's bad, you know that it's bad’. Every smoker knows it's bad for them. So I think you've got to make a team, ‘me and you, I'm here to help you’

Walter, 73: CS

Participants' experiences of stigmatization seemed to shape when participants felt smoking cessation support should be offered during lung cancer screening. Offering support at the beginning of the appointment was suggested to confirm perceived judgemental attitudes held by healthcare professionals where ‘people may think that I'll only be considered worthy of this investigative treatment if I'm a person who's going to agree to pack it in’ (Kathleen, 55: CS). Some described that this may prevent the attendee from engaging in screening at all. In comparison, an offer after receiving the lung cancer risk assessment, with the option of providing contact details for those who do not yet wish to access support within the initial appointment, was viewed as more appropriate. This made participants feel their autonomy would be respected and are not ‘forced’ to access support. Relatedly, participants felt that staff should only offer support once during the initial screening appointment; multiple offers within a single appointment were viewed as pressuring:

I mean, the thing is, I haven't forgotten since the appointment what was discussed, so therefore why would there be a need for you to reiterate it? […] I wouldn't be expecting somebody else to then reiterate what's already been conveyed to me […] it's like you're dictating, pressing this point.

Bernie, 55: CS

Participants expressed a need for ongoing support if an attendee takes up the within‐appointment cessation offer. This integrated approach was described as the service showing ‘genuine care’ for attendees, without making the offer feel like a ‘tick box’ exercise. To establish trust, having the same staff member for each cessation‐specific interaction was endorsed. Participants acknowledged that there may not always be the capacity to do so; however, continuity was expected to facilitate the development of a supportive relationship, increasing attendees' likelihood of sustained cessation following a quit attempt.

“Because, it's more personal, you know, it just feels like, you know, sometimes when you don't, you have to give all the information again and again and again […] seeing that same person will probably encourage more people to give up.

Patricia, 67: CS

2.5.1.3

*Theme 3: Creating an equitable service*

Participants reflected on previous quit attempts, with varied success. Some experienced becoming ‘lost in the system’ when trying to access cessation services. Despite seeking help, participants were not contacted by the service or were discharged without their knowledge. This led to a loss of confidence in services and subsequent disengagement:

I went to see my GP. He said ‘why [didn't] you go for this…?’ And so I explained to him, and he said, ‘well, no, you need to challenge that, because they've got you down here as you failed to turn up’. I said, ‘well, that's not the case’ […] I never bothered going back at all, if that's the attitude, I don't want to.

Len, 70: CS

In contrast, a ‘one stop shop’, where attendees can access immediate support within the screening appointment was viewed as ideal by many. This was also suggested to minimize pessimism about the efficacy of referrals and facilitate immediate rapport building with staff. Offering support immediately provides an opportunity to capitalize on the teachable moment that occurs within the screening appointment:

[…] you're on a bit of like a mission. So rather than go home and cool it off, especially if…like I say, we're all a bit of bravado, I don't think my lungs will be overly damaged because I'm fit and healthy. But once I was there and you're on the roll with it, yeah, I think it would be beneficial if it was offered there as well.

Helen, 56: CS

The desire for a person‐centred approach to smoking cessation was discussed as a key enabler for engagement. In addition to receipt of personalized lung health information as a gateway to an initial discussion, participants felt that cessation discussions and interventions should be tailored further. Participants often stated that there is no ‘one size fits all’ cessation method with many varied opinions on individual cessation methods, such as nicotine replacement or e‐cigarettes. Participants highlighted the need for multiple methods to be available throughout ongoing support, to allow attendees to find what is right for them:

Explain to them, 'you can try this, you can try that, you can try that, it's up to you, which one do you want to do? You know, and if it doesn't work, we can carry on' […] You know, don't just say, 'well, if that doesn't work, get out the door, you're off, there's no hope for you'.

Walter, 73: CS

In addition to the need for tailoring, ensuring accessible support was emphasized as important. Participants wished for attendees to be offered multiple modalities for ongoing support, wanting the choice of local, face‐to‐face (individual or group) or telephone support to account for the availability and possible practical barriers that attendees may have, such as work commitments or inability to travel due to financial or mobility difficulties:

if it's somewhere you can get to and it only takes half an hour to get there and back, that's brilliant. If they told me I had to go to [further away] or somewhere, I'd tell them where to go.

Anthony, 68: CS

## 3 DISCUSSION

This study explored attitudes towards and preferences for smoking cessation support and integration within lung cancer screening from the perspective of those potentially eligible to attend. Overall, ‘in the moment’ smoking cessation support was viewed as a fundamental part of lung cancer screening, where the provision of personalized risk‐based information can be a key motivator for cessation uptake. Participants highlighted the importance of offering a nonjudgemental, inclusive and accessible service to promote engagement.

Findings illustrate the potential for increased salience towards smoking behaviour after receiving personalized evidence of the impact of smoking on lung health. The introduction of a smoking cessation discussion at appropriate points during the screening pathway (e.g., initial appointment; LDCT results provision; investigation of suspicious or incidental findings) could increase participation or reaffirm reasons for stopping in people who have recently quit. Indeed, research has shown that abnormal spirometry results,^28^, ^41^ abnormal screening results including the need for further tests^18^, ^19^, ^28^ or a lung cancer diagnosis^42^ may lead to increased motivation and the likelihood of cessation. Additionally, the time between registering for lung cancer screening and receipt of results is associated with increased ‘readiness to quit’, particularly among individuals attending their first screening.^43^ In comparison, cessation discussions when deciding whether to have screening itself, before registering for lung cancer screening, has been viewed as unlikely to impact cessation from the perspective of both clinicians and individuals offered screening.^44^

The caveat of potential decreased motivation resulting from receiving an ‘all clear’ has also been identified as a concern among screening staff^45^ and the current study participants. There is currently no evidence to support that a ‘licence to smoke’ occurs in practice^46^ although a US‐based smoking cessation trial found that attrition was higher for participants who had negative LDCT scan results.^47^ Ongoing research will help to clarify the impact of the receipt of a personalized cessation discussion incorporating scan results alongside communication to support self‐efficacy and improved health consequences from cessation regardless of the type of result.^48^ A self‐help booklet intervention development project has also targeted negative results, with screening eligible individuals shaping a booklet section regarding ‘dodging the bullet’, discussing the dilemma faced by individuals with a negative screening result regarding smoking cessation.^49^

Fatalism was also acknowledged as a potential response to considering quitting, which has been previously identified, alongside the low perceived efficacy of smoking cessation in reducing the risk of lung cancer, as a barrier to lung cancer screening engagement among eligible individuals in the United Kingdom.^29^, ^50^, ^51^, ^52^, ^53^ The present study extends these findings by demonstrating that fatalism may also influence those who have already made the decision to attend, acting as a barrier to cessation uptake. Discussing the benefits of cessation regardless of age, current health or smoking history with attendees may increase intention to quit. For example, even following the diagnosis of lung cancer, smoking cessation is associated with reduced progression and mortality across cancer stages indicating that it is never ‘too late’ to consider quitting.^54^ The adoption of an ‘opt‐out’ service delivery model for discussion of smoking cessation would ensure all attendees are able to discuss their views surrounding risk and has been shown to improve cessation uptake.^55^

Previous research has illustrated that individuals eligible for lung cancer screening report smoking‐associated external and internalized stigma.^29^, ^56^ The present study builds on this by exploring how preferences for integration of smoking cessation within lung cancer screening are shaped by these views. Judgemental communication styles by healthcare professionals may reinforce smoking behaviour and reduce motivation to quit. Participants in the present study demonstrated some avoidance and mistrust of healthcare professionals consistent with prior work demonstrating that an expectation of judgement can deter prospective attendees from screening.^24^ In contrast, receiving person‐centred support from a healthcare professional facilitates autonomy. Consistent with prior work,^57^ tailoring communication to the needs of screening attendees was emphasized as vital for smoking cessation uptake. Lung cancer screening invitations should emphasize a nonjudgemental approach to prevent nonattendance due to expected stigmatization. Screening staff may benefit from communication skills training to promote engagement in both cessation and future screening rounds.^58^ This may include staff acknowledging the difficulty of smoking cessation, discussing smoking in a sensitive and empathic manner, and framing *how* attendees are asked if they would like to access cessation support. The setting of a lung cancer screening appointment was discussed by participants as increasing consideration of cessation, in comparison to a more general setting such as a GP appointment. However, what is not known is whether attending a setting specifically tailored for individuals who smoke, decreases feelings of stigmatization. Further work should investigate this among screening attendees.

Participants emphasized the importance of flexible and accessible services. Existing evidence has demonstrated that the locality of screening services is an important determinant of screening uptake.^59^ The present study confirms that convenience and locality of smoking cessation support are also important facilitators for a screening eligible population. In contrast to brief interventions, which have been predominantly provided within UK‐based lung cancer screening research (e.g., National Centre for Smoking Cessation and Training's Very Brief Advice),^60^, ^61^ a ‘one stop shop’ where attendees can initiate engagement in smoking cessation services within the screening appointment was highlighted as an enabler to cessation uptake. Indeed, attendees within the Italian lung screening trial (ITALUNG) receiving screening at a centre with integrated smoking cessation had greater odds of cessation compared to attendees at other screening centres.^62^ Additionally, a trial in England has shown that the provision of immediate smoking cessation within a TLHC is associated with an increase in quit rates at a 3‐month follow‐up.^63^ Integrating cessation interventions within the screening appointment and disclosure of screening results may increase cessation uptake by ensuring accessibility of the service, providing readily available treatment, and preventing referral‐related disengagement. However, the ability to integrate is largely dependent on the model of lung cancer screening service delivery. Yet, this remains an important consideration as poor referral processes, and appointment delays are significant barriers to cessation service uptake.^57^

The need for tailoring and flexibility of services regarding ongoing support modality (face‐to‐face, telephone, online), treatment method (e.g., nicotine replacement products, medications, e‐cigarettes and individual or group support) and discussion content (e.g., exploring and debunking any myths regarding cessation that an attendee has concerns about) were emphasized by participants as key to creating acceptable, effective cessation services. Indeed, the ability to provide tailored, multimodal cessation interventions has been shown to potentially support smoking cessation among older individuals who smoke, from deprived backgrounds, many of whom may be eligible for lung cancer screening.^64^ The ability to be flexible has also been identified as an important facilitator of successful implementation of smoking cessation services within hospitals,^65^ and appears to also be important within a lung cancer screening context. However, the setup and commissioning of UK smoking cessation services within public health (where each local authority commissions its own cessation services) may limit the scope of what lung cancer screening services are able to offer. For example, as of 2021, only 76% of surveyed local authorities in England offer a specialist stop smoking service.^66^

The use of the TDF^30^, ^31^ for data analysis allows specific theoretical components to be identified, which can be targeted by subsequent staff‐ and attendee‐centred interventions. For example, providing attendees with personalized information regarding their lung health, including further information after screening results, may increase the ‘perceived consequences’ of smoking, thus encouraging cessation uptake. Staff training centred around ‘social influences’ of cessation discussions (e.g., prior stigmatization, need for an empathic approach) may promote appropriate communication styles among staff. Additionally, interventions containing components aiming to increase self‐efficacy, and positively ‘reinforcing’ quit attempts may assist attendees to overcome the low ‘belief in capabilities’ held by some participants regarding smoking cessation.

To the research team's knowledge, this is the first qualitative study conducted in the UK, which investigates attitudes towards and preferences for cessation delivery as part of lung cancer screening. Using qualitative methods facilitated the collection of rich data, including unique insights for inclusion in clinical guidelines and for service development. The community engagement strategy facilitated the recruitment of individuals in areas of high deprivation, including those without access to computers. Additionally, we recruited individuals with a wide range of educational achievement, and smoking histories, reflecting the target audience of screening‐eligible individuals. Although adaptation to social media recruitment allowed data collection to continue during the first COVID‐19 lockdown, it also meant that individuals without internet access could not be recruited. The final sample was also not diverse with regard to race and ethnicity. Future research should include purposive sampling across races and ethnicities to reflect diversity in screening active areas as international research has shown racial and ethnic disparities are prevalent across the lung cancer screening pathway, for example, eligibility, uptake and follow‐up care.^67^ Finally, throughout interviews, participants who had RQS largely discussed how cessation support could be provided to attendees who smoke, rather than those who had previously quit. Future research could explore the role that lung cancer screening may play in relapse prevention among individuals who previously smoked, regardless of eligibility for LDCT scanning.

## 4 CONCLUSION

To conclude, integrating smoking cessation within lung cancer screening was viewed by those eligible as necessary and expected, regardless of smoking status and plans to quit. The ability of lung cancer screening to provide attendees with personalized information regarding the impact of smoking on their health was viewed as a key factor affecting the potential uptake of smoking cessation. A non‐judgemental, accessible and inclusive service, which addresses patient‐level barriers, such as fatalism, anxiety, and avoidance provides a unique opportunity to engage attendees in smoking cessation.

## AUTHOR CONTRIBUTIONS

All authors meet the four criteria for ICMJE authorship. Presented below is each author's contribution relative to CRediT Classification: **Samantha Groves**: data curation, formal analysis (lead), project administration (equal), visualization (lead), writing (original draft, lead) and writing – reviewing and editing (equal). **Grace McCutchan**: Conceptualization, funding acquisition, formal analysis/interpretation, materials, writing – reviewing and editing (equal). **Samantha L. Quaife**: Conceptualization, funding acquisition, methodology, materials, interpretation, writing – reviewing and editing (equal). **Rachael L. Murray**: Conceptualization, funding acquisition, methodology, materials, writing – reviewing and editing (equal). **Jamie S. Ostroff**: Conceptualization, funding acquisition, methodology, interpretation, writing – reviewing and editing (equal). **Kate Brain**: Funding acquisition, methodology, methodology, supervision, writing – reviewing and editing (equal). **Philip A. J. Crosbie**: Conceptualization, funding acquisition, materials, writing – reviewing and editing (equal). **Janelle Yorke**: Conceptualization, funding acquisition, methodology, writing – reviewing and editing (equal). **David Baldwin**: Funding acquisition, supervision, writing – reviewing and editing (equal). **John K. Field**: Funding acquisition, supervision, writing – reviewing and editing (equal). **Lorna McWilliams**: Conceptualization (lead), funding acquisition (lead), formal analysis, project administration (equal), methodology (lead), visualization, writing (original draft) and writing – reviewing and editing (equal).

### CONFLICTS OF INTEREST

The authors declare no conflicts of interest.

## Supporting information

Supplementary information.

## Data Availability

The data that support the findings of this study are available from the corresponding author upon reasonable request.
