# Supplementary material for: Attitudes towards the integration of smoking cessation into lung cancer screening in the United Kingdom: A qualitative study of individuals eligible to attend
Source: Health Expect. 2022 May 5;25(4):1703–16. doi: 10.1111/hex.13513 (PMC9327806; doi:10.1111/hex.13513)
Supplement: Supplementary file 1 — Supplementary information. [file HEX-25--s001.docx]

**Supplementary File 1**

**Sample Topic Guide**

**Prologue:**

In the UK, some research studies and areas are testing to see if lung health checks/ lung cancer screening (use phrase as appropriate) should be made available through the NHS (like the breast and bowel cancer screening currently is). Lung health checks involve [*area specific process for local lung health check explained and* *give leaflet if available for each screening area and information about the service].* It also might include being asked about whether you smoke and if you would like support to stop smoking. Remember that I am not asking you to stop smoking today.

We would like your feedback/advice on the best ways for staff to give patients stop smoking support as part of lung health checks. I’m going to ask some questions about how you think stop smoking support could be given to people who go to lung screening and have a lung health check. I am interested in what you think: both positive and negative, and there are no right or wrong answers.

**Sample Questions**

‘I’d like you to imagine you have been invited to take part in a lung health check, and the service offers advice to help you stop smoking [*remind group about specific local context and what the lung screening offers in the local area*]’

1. What do you think about the lung health check service offering stop smoking support?

1. How would you feel about being offered stop smoking support as part of lung health checks?

1. When should stop smoking support be offered at a lung health check appointment?

[*Potential probes*: at the beginning, during or end; not at all]

1. Who do you think would be best to discuss stop smoking support at lung health check services?

[*Potential probes*: receptionist, nurse, scanner, doctor, GP]

Why do you think that?

What would make it easy for attendees if it was that staff member(s)?

What would make it hard for attendees if it was that staff member(s)?

1. How would you prefer to receive stop smoking advice in a lung health check service appointment?

[*Potential probes*: ask whether someone feels ready to stop smoking; ask whether someone wants some advice to stop smoking; offer face-to-face support at the screening appointment; offer written stop smoking information to take home; offer stop smoking medication; give referral information about local stop smoking support; e-cigarettes]

For each aspect of support:

Why do you think that?

What would make it easy for them?

What would make it hard?

1. Is there anything else you would like to add? Is there anything we were going to discuss but haven’t? Any other thoughts about the questions we’ve discussed?
